# Hypoxia-Stabilized HIF1α Restricts Hair Cell Reprogramming by Suppressing Wnt Signaling in the Mammalian Cochlea

**DOI:** 10.3390/cells15161440

**Published:** 2026-08-11

**Authors:** Qing Liu, Yuanning Guo, Xinyu Wang, Shu Wang, Ao Li, Guoqiang Wan

**Affiliations:** 1 Ministry of Education Key Laboratory of Model Animal for Disease Study, Department of Otolaryngology Head and Neck Surgery, Jiangsu Provincial Key Medical Discipline (Laboratory), the Affiliated Drum Tower Hospital of Medical School and the Model Animal Research Center of Medical School, Nanjing University, Nanjing 210008, China; liuqing@njglyy.com (Q.L.); guoyn@smail.nju.edu.cn (Y.G.); 502023350037@smail.nju.edu.cn (X.W.); 502024350049@smail.nju.edu.cn (S.W.); 2 Research Institute of Otolaryngology, No. 321 Zhongshan Road, Nanjing 210008, China; 3 Jiangsu Key Laboratory of Molecular Medicine, National Resource Center for Mutant Mice of China, Nanjing University, Nanjing 210008, China

**Keywords:** hair cell reprogramming, HIF1α, hypoxia, Wnt signaling, cochlea, hearing loss

## Abstract

**Highlights:**

**What are the main findings?**

- HIF1α accumulates in the intact cochlea and suppresses hair cell reprogramming.
- Hypoxia mimics the in vivo block to Wnt- dependent hair cell reprogramming.

**What are the implications of the main findings?**

- HIF1α selectively inhibits Wnt signaling.
- HIF1α inhibitors restore hair cell reprogramming, offering a translational strategy for hearing loss.

**Abstract:**

Sensorineural hearing loss, caused by irreversible hair cell (HC) loss, remains incurable due to the inability of the mammalian cochlea to regenerate HCs spontaneously. Although the combinatorial modulation of Notch and Wnt signaling robustly reprograms cochlear supporting cells to HCs in vitro, these strategies show limited success in vivo, which suggests that the native cochlear microenvironment imposes inhibitory cues. Here, we identify hypoxia-inducible factor 1α (HIF1α) as a critical barrier to HC reprogramming in vivo. We found that HIF1α accumulates in the basilar membrane of the intact cochlea, whereas ex vivo culture rapidly eliminates this accumulation. Mimicking the in vivo hypoxic state recapitulated the reprogramming blockade, hindering supporting cells from completing the fate transition to HCs. Mechanistically, HIF1α selectively suppressed Wnt signaling, as evidenced by the reduced expression of Wnt target genes and Wnt10a. Importantly, the pharmacological inhibition of HIF1α rescued the impaired reprogramming under hypoxia. These findings demonstrate that HIF1α accumulation in the hypoxic cochlear environment antagonizes Wnt-mediated HC differentiation, providing a mechanistic explanation for the failure of in vivo regeneration. Targeting HIF1α to restore supporting cell reprogramming competence within the hypoxic cochlear niche represents a promising, clinically translatable strategy for functional HC regeneration and hearing restoration.

## 1. Introduction

Sensorineural hearing loss (SNHL) is the most prevalent sensory disorder worldwide, affecting an estimated 1.5 billion people, and is primarily caused by the irreversible loss of cochlear hair cells (HCs) due to acoustic trauma, aging, or ototoxic drugs [1]. Unlike their counterparts in non-mammalian vertebrates, mammalian HCs lack the intrinsic capacity for spontaneous regeneration following damage, which renders hearing loss a permanent condition [2]. Given the societal and economic burden of hearing impairment, there is an urgent need to develop therapeutic strategies that can effectively regenerate functional HCs in vivo.

The cochlear sensory epithelium contains multiple subtypes of supporting cells, including pillar cells, Deiters’ cells, and inner phalangeal cells, which serve as the principal cellular substrate for regenerative reprogramming strategies in the postnatal cochlea [3]. Under defined combinatorial signaling conditions, these cells retain the capacity to undergo fate transition toward the hair cell lineage, a process driven by Wnt/β-catenin activation in combination with Notch inhibition. Specifically, Notch inhibition (e.g., DAPT) promotes supporting cell-to-HC reprogramming in neonatal cochlear explants [4,5]. Meanwhile, Wnt activation (e.g., CHIR99021) expands Lgr5^+^ supporting cells and synergizes with Notch blockade to generate HCs in organoids and explants [6,7,8,9,10]. However, these regimens consistently fail in vivo, which indicates that cell-intrinsic or niche-derived signals actively suppress reprogramming competence in the intact organ.

Oxygen tension is one such signal. The inner ear operates at a perilymphatic pO_2_ of 2–5%, much lower than atmospheric oxygen [11]. This physiological hypoxia is sensed by hypoxia-inducible factor 1α (HIF1α), a transcription factor stabilized under low oxygen and degraded under normoxia via VHL. Under hypoxic conditions, HIF1α stabilizes, translocates to the nucleus, and drives the expression of hundreds of target genes involved in metabolism, angiogenesis, and cell survival [12]. Beyond its classical metabolic roles, HIF1α has emerged as a conserved regulator of stem and progenitor cell quiescence across multiple tissue systems. In hematopoietic stem cells, neural stem cells, and muscle satellite cells, physiological hypoxia stabilizes HIF1α to maintain progenitors in a quiescent, non-differentiating state, a protective mechanism under homeostasis that can become a barrier to regeneration following injury [13,14,15]. This quiescence-enforcing function frequently operates through intersection with major fate-determining pathways, including Wnt/β-catenin, which HIF1α has been shown to suppress through competition for transcriptional co-activators or the induction of pathway antagonists [14,16,17]. These findings in other systems support a plausible hypothesis but do not constitute direct evidence in the cochlea. Whether HIF1α similarly suppresses reprogramming in cochlear supporting cells remains untested. The present study directly investigates this question.

In this study, we investigated whether HIF1α accumulation in the in vivo cochlea antagonizes Wnt-mediated HC reprogramming. By comparing in vivo models with in vitro explants and organoids under normoxia and hypoxia, we examined the role of HIF1α in this process. Our findings suggest that HIF1α may act as a previously underappreciated barrier to Wnt-mediated HC differentiation and that targeting this pathway could offer a potential strategy for enhancing HC regeneration.

## 2. Materials and Methods

### 2.1. Experimental Design Overview

This study investigated the role of HIF1α in cochlear hair cell (HC) regeneration using complementary experimental systems. The overall workflow comprised: (1) in vivo regeneration assays using genetic mouse models to evaluate HC reprogramming following Wnt/Notch modulation; (2) in vitro cochlear explant and 3D organoid cultures under normoxia (21% O_2_) or physiological hypoxia (5% O_2_) to dissect the cellular and molecular mechanisms; and (3) molecular analyses including immunofluorescence, Western blotting, and RT-qPCR to assess HIF1α accumulation, Wnt/Notch pathway activity, and HC reprogramming outcomes. A schematic summary of the experimental design is provided in Figure S1.

### 2.2. Reagents and Primers

All details of chemicals, primers, antibodies and other reagents are listed in Table S1.

### 2.3. Mouse Strains and Mouse Experiments In Vivo

Mouse strains: Sox2-CreER (#017593) mice were purchased from the Jackson Laboratory. β-catenin mice were kindly provided by Dr Zhiyong Liu. Wildtype C57BL6 mice and Rosa26-CAG-STOP-Cas9-tdTomato (Rosa26-tdTomato) mice were purchased from Gempharmatech (Nanjing, China). Both female and male C57BL6 mice were used and randomly assigned in this study. Mice were housed under specific pathogen-free (SPF) conditions with a 12 h light/dark cycle and ad libitum access to food and water. All animal procedures were approved by the Institutional Animal Care and Use Committee of Model Animal Research Center of Nanjing University, China, with the approval number #WGQ06.

Pharmacological regeneration in vivo: For in vivo regeneration experiments, we used Rosa26-tdTomato mice that received continuous intraperitoneal administration of either CHIR (S1264, Selleck, Houston, TX, USA) alone or CHIR combined with DAPT (sc-201315, Santa Cruz Biotechnology, Dallas, TX, USA) for 14 d following tamoxifen (T5648, Sigma, St. Louis, MO, USA) treatment (50 mg/kg in corn oil) at P1–P2 to induce Cre-mediated tdTomato labeling in Sox2-expressing supporting cells. Following tamoxifen induction, mice received continuous intraperitoneal administration of either CHIR99021 alone (30 mg/kg) or CHIR99021 combined with DAPT (20 mg/kg) for 14 consecutive days (once daily). The compounds were dissolved in a vehicle of 10% DMSO (D8418, Sigma) and 90% corn oil; drug solutions were administered at a dosing volume of 100 μL per 10 g of body weight. Control animals received an equal volume of vehicle alone on the same schedule. At P17, mice cochleae were harvested for wholemount immunofluorescence analysis.

AAV-mediated β-catenin overexpression: For β-cantein-overexpressing mice, hair cell numbers were assessed at either P18 or P29 following round window injection of AAV-Cre at P2.AAV-Cre (serotype IE, titer 1 × 10^13^ vg/mL, 1.5 μL) into the scala tympani through the round window membrane using a glass micropipette attached to a microsyringe pump (0.1 μL/min infusion rate).

### 2.4. 3D Cochlear Organoid Culture

Cochlear organoid culture was carried out as described in our previous studies [3,18]. Briefly, cochlear sensory epithelia tissues were dissected from postnatal day 3 (P3) mice and were digested sequentially with thermolysin (P1512, Sigma) and TrypLE (12604013, Thermo Fisher Scientific, Waltham, MA, USA) to ascertain single cells. Single cells were filtered and cultured in 96-well plate with ultra-low attachment surface in the culture medium with 0.02 mg/mL laminin (354232, Corning Incorporated, Corning, NY, USA) and 2% Matrigel (40191ES08, Yeasen, Shanghai, China). The culture media (DMEM and F12 in 1:1 ratio) (12634010, Thermo) were supplemented with Glutamax I, N2, B27, murine FGF (50 ng/mL) (450-33, PeproTech, Cranbury, NJ, USA), murine EGF (50 ng/mL) (315-09, PeproTech), murine IGF-1 (50 ng/mL) (250-19, PeproTech) and small molecules, including CHIR99021, pVc (49752, Sigma), VPA (676380, Sigma)and 616452 (S7223, Selleck). Organoids were induced to differentiate at DIV10 (day 10 in vitro) in medium consisting of DMEM/F12 supplemented with Glutamax I (35050061, Thermo), B27 (12587010, Thermo), N2 (17502048, Thermo) and small molecules, including CHIR99021, Regorafenib or LY411575 (209984-56-5, Santa Cruz). For hypoxia culture conditions, organoids were cultured in an incubator equilibrated with 5% O_2_.

### 2.5. Cochlear Explant Culture

Cochlear explant culture was performed as previously described [3,18]. Briefly, cochlear sensory epithelia from P3 mice were dissected and cultured in a 35 mm collagen-coated dish. The explants were maintained in DMEM/F12 media supplemented with Glutamax I, B27, N2 and Penicillin G (P3032, Sigma). After 1 d, the explants were cultured in medium without growth factors but with CHIR99021 (3 µM) and small molecules (DAPT, Regorafenib, 2-MeOE (S1233, Selleck), PX478 (S7612, Selleck) and CoCl_2_ (232696, Sigma). Fresh media were replaced every other day until sample collection and further analyses. For hypoxia culture conditions, cochlear explants were cultured in an incubator equilibrated with 5% O_2_.

Regarding the neomycin damage experiments of *β-catenin:Sox2-CreER* mice, cochlear explants were maintained in DMEM/F12 medium supplemented with Glutamax I, B27, N2 and Penicillin G, in the presence of 0.15 M neomycin. After 1 d of neomycin damage, the medium was changed to induction medium containing CHIR99021 (3 µM), (z)-4-Hydroxytamoxifn (3412, Tocris, Minneapolis, MN, USA) and DAPT or Regorafenib.

### 2.6. Immunofluorescence

Immunofluorescent analyses of cochlear organoids and explants were performed as previously described [3,18]. Cochlear explants or organoids were fixed with 4% paraformaldehyde (PFA) in PBS for 0.5 h. Permeabilization and blocking were carried out simultaneously in the blocking buffer consisting of 0.3% Triton X-100 (Sangon Biotech, Shanhai, China) in 5% heat-inactivated normal horse serum (NHS) (26050088, Thermo) in PBS for 1 h at RT. Primary and secondary antibodies were prepared in 0.3% Triton X-100 and 1% heat-inactivated NHS. Hair cells were immunostained with hair cell markers, including Pou4f3 (sc-81980, Santa cruz) and Myo7a (25-6790, Proteus Biosciences, Ramona, CA, USA). For immunofluorescence of the murine cochlear tissues, temporal bones were extracted, perfusion-fixed with 4% PFA at RT for 2 h, and then decalcified with 5% EDTA. Cochlear wholemounts were obtained by micro-dissection, blocked and immunolabeled with Pou4f3 and Myo7a. The confocal imaging was performed with Leica SP5 confocal microscope (Leica, Germany).

### 2.7. Western Blot Analyses

Total protein lysates from two P3 cochlear tissues or two cochlear explants were extracted in 20 µL lysis buffer (50 mM Tris-HCl, pH 7.4, 1% Triton X-100, 150 mM NaCl, 1 mM EDTA) with 1 mM PMSF (Sangon Biotech, Shanghai, China) and the proteinase inhibitor cocktail (B14004, Selleck). The total lysates were freeze-thawed and heated at 95 °C in the loading buffer for 20 min. Samples were then electrophoresed in 10% SDS-PAGE and subjected to Western blot analysis with antibodies against HIF1α (36169, Cell Signaling Technology, Danvers, MA, USA) and β-actin (Actin) (4970, Cell Signaling Technology). Signals were visualized by the infrared imaging system (Tanon, Shanghai, China). Densitometry of the blot images was analyzed using the ImageJ software (version 1.46r, NIH, MD).

### 2.8. RNA Extraction and RT-QPCR

Cochlear organoids and explants were collected in 1.5 mL centrifuge tubes and digested in 1 mL RNAiso Plus (9109, Takara Bio, Kusatsu, Japan). Reverse transcription (RT) of the isolated total RNA was performed using the PrimeScript^™^ RT reagent Kit (RT101, Vazyme, Nanjing, China). QPCR was performed using the Hieff^®^ qPCR SYBR^®^ Green Master Mix (No Rox) kit (11201ES, Yeasen, Shanghai, China). *Gapdh* served as the reference gene. Sequences of the primers are specified in Table S1.

### 2.9. Statistical Analysis

Statistical analyses were performed using the Graphpad Prism 8 software (San Diego, CA, USA). Results are shown either as mean ± SD or mean ± SEM. Replicated values were obtained from 3–8 independent biological replicates. Each biological sample was allocated randomly to either control or treatment groups. Details of the statistical tests in each experiment are specified in figure legends. Sample sizes were determined empirically according to previous experience in the research field.

## 3. Results

### 3.1. Impaired Hair Cell Reprogramming In Vivo Is Associated with HIF1α Accumulation

**Figure 1:**
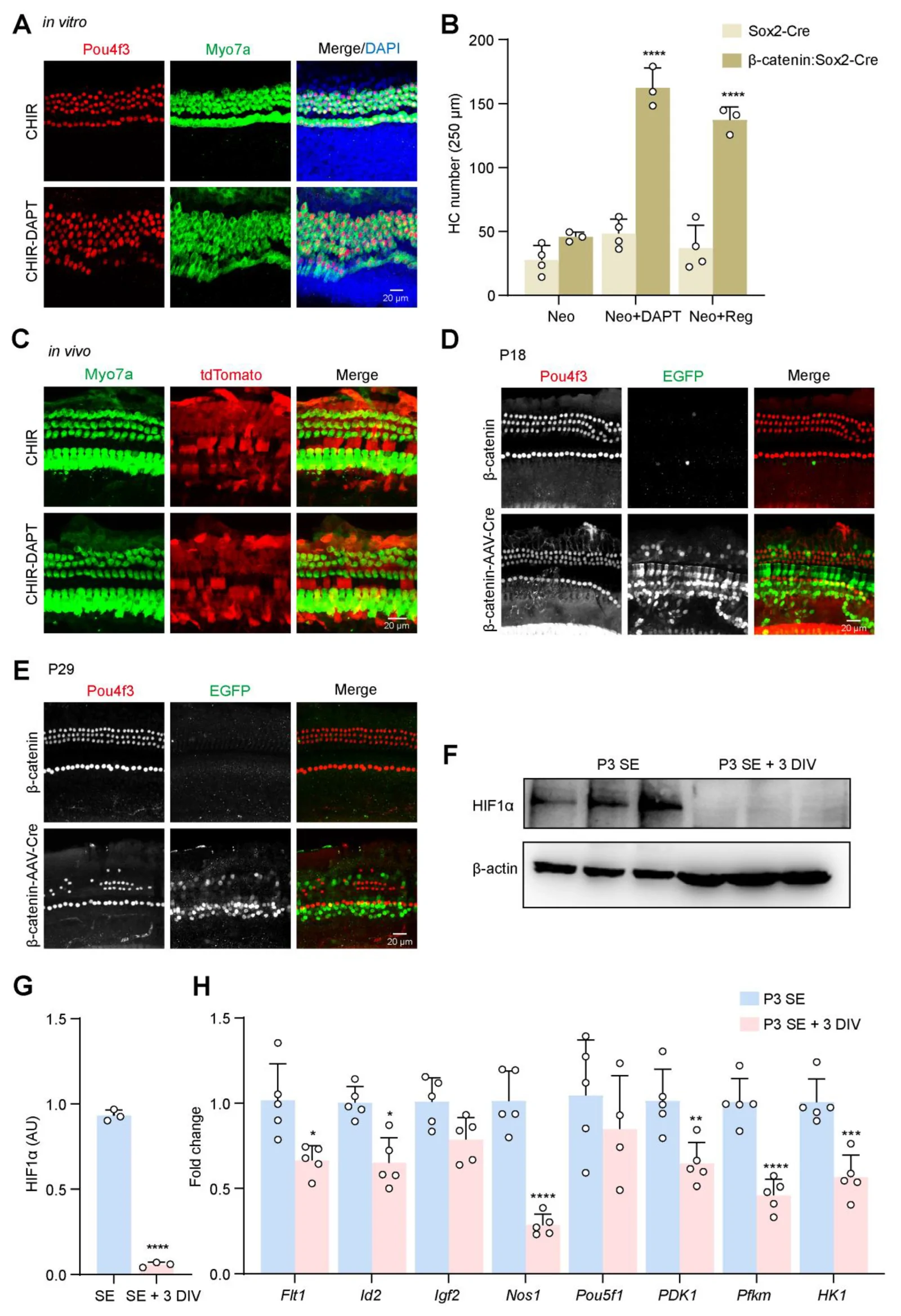
Hair cell reprogramming was not achieved in vivo and leads to HIF1α accumulation. (**A**) Inhibition of Notch signaling by DAPT promoted hair cell reprogramming in cochlear explants from P3 mice. Confocal images of cochlear explants treated with CHIR99021 or co-treated with DAPT. (Scale bars: 20 μm.) (**B**) Co-treatment with Reg or DAPT promoted hair cell reprogramming following β-catenin overexpression following in vitro damage by neomycin. *n* = 3–4 cochlear explants. Error bars represent mean ± SD. **** *p* < 0.0001 by one-way ANOVA. (**C**) Inhibition of Notch signaling by DAPT did not promote hair cell reprogramming in vivo. Confocal images of mice treated with CHIR99021 or co-treated with DAPT in vivo. (Scale bars: 20 μm.) (**D**) In vivo overexpression of β-catenin did not promote hair cell reprogramming in P18 mice. Confocal images of P18 mice treated with PBS or AAV-Cre. (Scale bars: 20 μm.) (**E**) Sustained in vivo overexpression of β-catenin led to hair cell death in P29 mice. Confocal images of P29 mice treated with PBS or AAV-Cre. (Scale bars: 20 μm.) (**F**) HIF1α accumulation was observed in the basilar membrane in vivo, as determined by Western blot comparison between tissue collected at P3 and tissue cultured ex vivo for 3 d starting from P3. (**G**) Densitometric quantification of the Western blot analysis from (**F**). Error bars represent mean ± SEM. *n* = 3 independent experiments. **** *p* < 0.0001 by one-way ANOVA. (**H**) RT-qPCR analyses of HIF1α target genes comparing in vivo and in vitro conditions. Error bars represent mean ± SD. *n* = 5 biological replicates. * *p* < 0.05, ** *p* < 0.01, *** *p* < 0.001 and **** *p* < 0.0001 by *t*-test.

To understand why the robust HC reprogramming observed in vitro fails to translate in vivo, we first compared the effects of Notch signaling inhibition on hair cell reprogramming in vitro and in vivo. In cochlear explants from postnatal day 3 (P3) mice, the inhibition of Notch signaling with DAPT promoted hair cell reprogramming from supporting cells, as did treatment with CHIR99021 or co-treatment with DAPT (Figure 1A). Quantitative analysis confirmed that co-treatment with DAPT or Regorafenib, a VEGFR inhibitor previously reported to promote HC reprogramming [3], significantly promoted hair cell reprogramming following β-catenin overexpression under in vitro neomycin-damage conditions (Figure 1B). In striking contrast, the inhibition of Notch signaling by DAPT failed to promote hair cell reprogramming in vivo (Figure 1C). Similarly, in vivo overexpression of β-catenin did not promote hair cell reprogramming in P18 mice (Figure 1D). Moreover, sustained in vivo overexpression of β-catenin led to hair cell death in P29 mice (Figure 1E), which suggests that the in vivo environment imposes constraints on regenerative capacity that are not present in vitro. These results prompt us to investigate the endogenous inhibitory factors that constrain regeneration specifically in the native cochlear environment.

To identify potential factors limiting hair cell reprogramming in vivo, we examined HIF1α levels. Western blot analysis revealed significant HIF1α accumulation in the basilar membrane in vivo compared to tissue cultured ex vivo for 3 days starting from P3 (Figure 1F,G). RT-qPCR analyses further demonstrated the upregulation of HIF1α target genes in in vivo compared to in vitro conditions (Figure 1H). These findings indicate that HIF1α accumulation in the in vivo cochlear environment may contribute to the failure of hair cell reprogramming.

### 3.2. Hypoxia Suppresses Wnt-Driven Supporting Cell Reprogramming Without Affecting Hair Cell Survival

**Figure 2:**
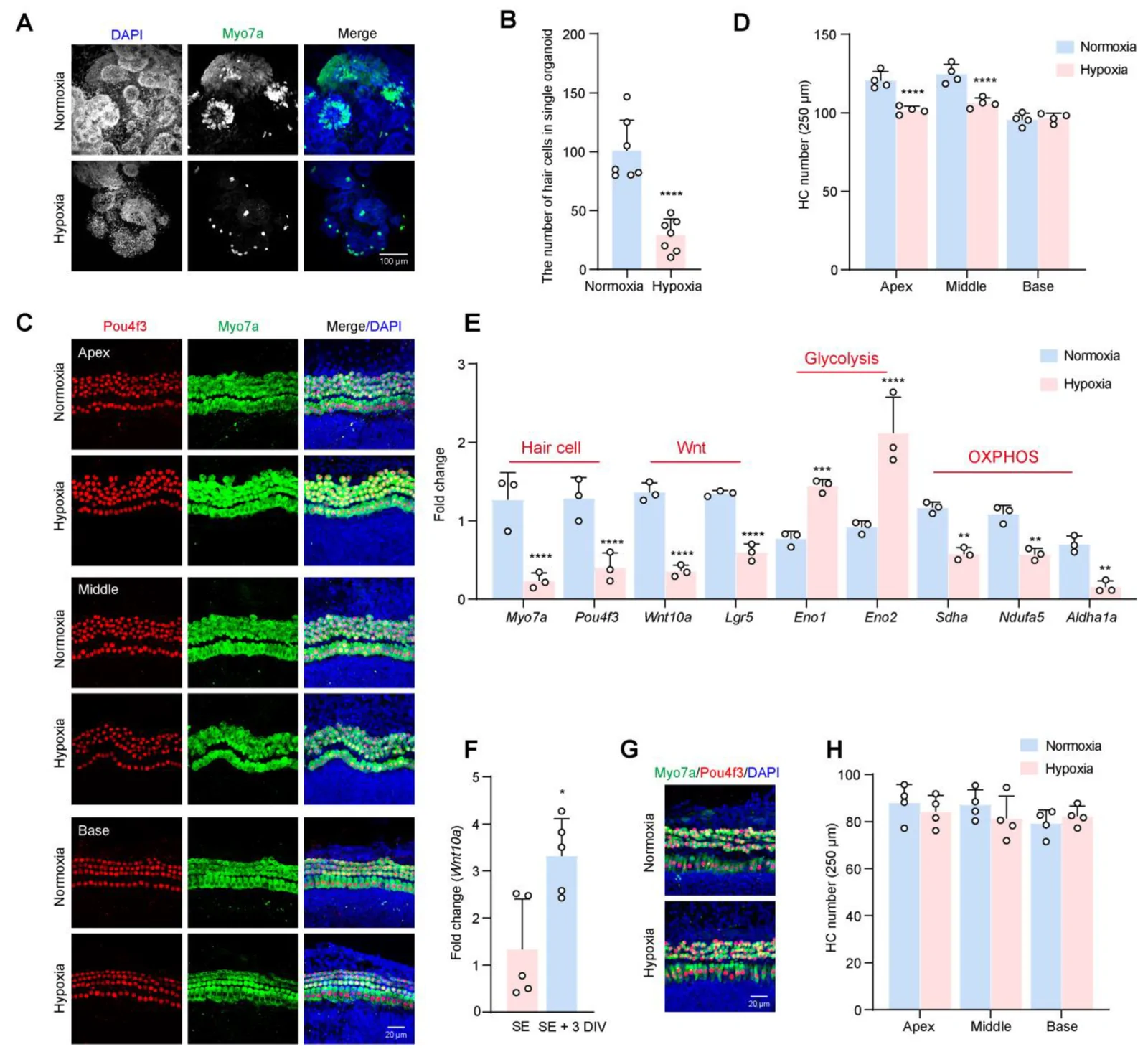
Hair cell reprogramming efficiency was reduced under in vitro hypoxia conditions, while survival remained unaffected. (**A**,**B**) Hypoxia (5% O_2_) treatment reduced the LY411575-induced increase in hair cell numbers. (**A**) Confocal images of differentiated cochlear organoids. (Scale bars: 20 μm.) Data are presented as the average number of hair cells per organoid, with each sample consisting of 100 organoids. (**B**) Error bars represent mean ± SEM. *n* = 7 biological replicates, with each replicate containing 100 organoids. **** *p* < 0.0001 by one-way ANOVA. (**C**,**D**) Hypoxia (5% O_2_) treatment inhibited Regorafenib-induced hair cell reprogramming in the apex and middle regions of cochlear explants. (**D**) Error bars represent mean ± SD. *n* = 4 cochlear explants in the apex, middle, and base regions in each condition. **** *p* < 0.0001 by one-way ANOVA. (**C**) Confocal images of cochlear explants. (Scale bars: 20 μm.) (**E**) RT-qPCR analyses of hair cell, Wnt, glycolysis, and OXPHOS markers under normoxia and hypoxia. Error bars represent mean ± SD. *n* = 3 biological replicates, with each replicate containing 150–300 organoids. ** *p* < 0.01, *** *p* < 0.001 and **** *p* < 0.0001 by *t*-test. (**F**) RT-qPCR analyses of *Wnt10a* comparing in vivo and in vitro cochlear tissues. Error bars represent mean ± SD. *n* = 5 biological replicates. * *p* < 0.05 by *t*-test. (**G**,**H**) Hypoxia conditions did not affect hair cell survival. (**G**) Confocal images of cochlear explants. (Scale bars: 20 μm.) Quantification of hair cells in distinct regions of the cochlea. (**H**) Error bars represent mean ± SD. *n* = 4 cochlear explants in each condition.

Given the observed HIF1α accumulation in vivo, we next asked whether this biochemical feature directly contributes to the impaired reprogramming phenotype. To address this, we established hypoxic culture conditions (5% O_2_) to mimic the in vivo oxygen environment and examined its effect on HC differentiation in two complementary in vitro systems: 3D organoids and cochlear explants. The exposure of cochlear organoids to hypoxia (5% O_2_) significantly reduced the LY411575-induced increase in hair cell numbers (Figure 2A). Quantitative analysis revealed that hypoxia treatment markedly decreased hair cell reprogramming efficiency, as assessed by the average number of hair cells per organoid (Figure 2B). Similarly, hypoxia (5% O_2_) treatment inhibited Regorafenib-induced hair cell reprogramming in cochlear explants, particularly in the apex and middle regions (Figure 2C,D). Hypoxia, thus, recapitulates the in vivo reprogramming block in both systems, which validates it as an in vitro surrogate for the cochlear niche.

RT-qPCR analyses revealed that hypoxia downregulated hair cell markers, Wnt signaling components, and OXPHOS markers but upregulated glycolysis markers compared to normoxia (Figure 2E). Notably, *Wnt10a* expression was significantly lower in in vivo compared to in vitro conditions (Figure 2F), which suggests that Wnt signaling may be compromised under hypoxic conditions. Importantly, hypoxia conditions did not affect hair cell survival, as evidenced by the quantification of hair cells in distinct regions of the cochlea (Figure 2G,H). These results indicate that hypoxia specifically impairs hair cell reprogramming rather than inducing cell death.

### 3.3. HIF1α Accumulation Inhibits Hair Cell Reprogramming Through Wnt Signaling Modulation

**Figure 3:**
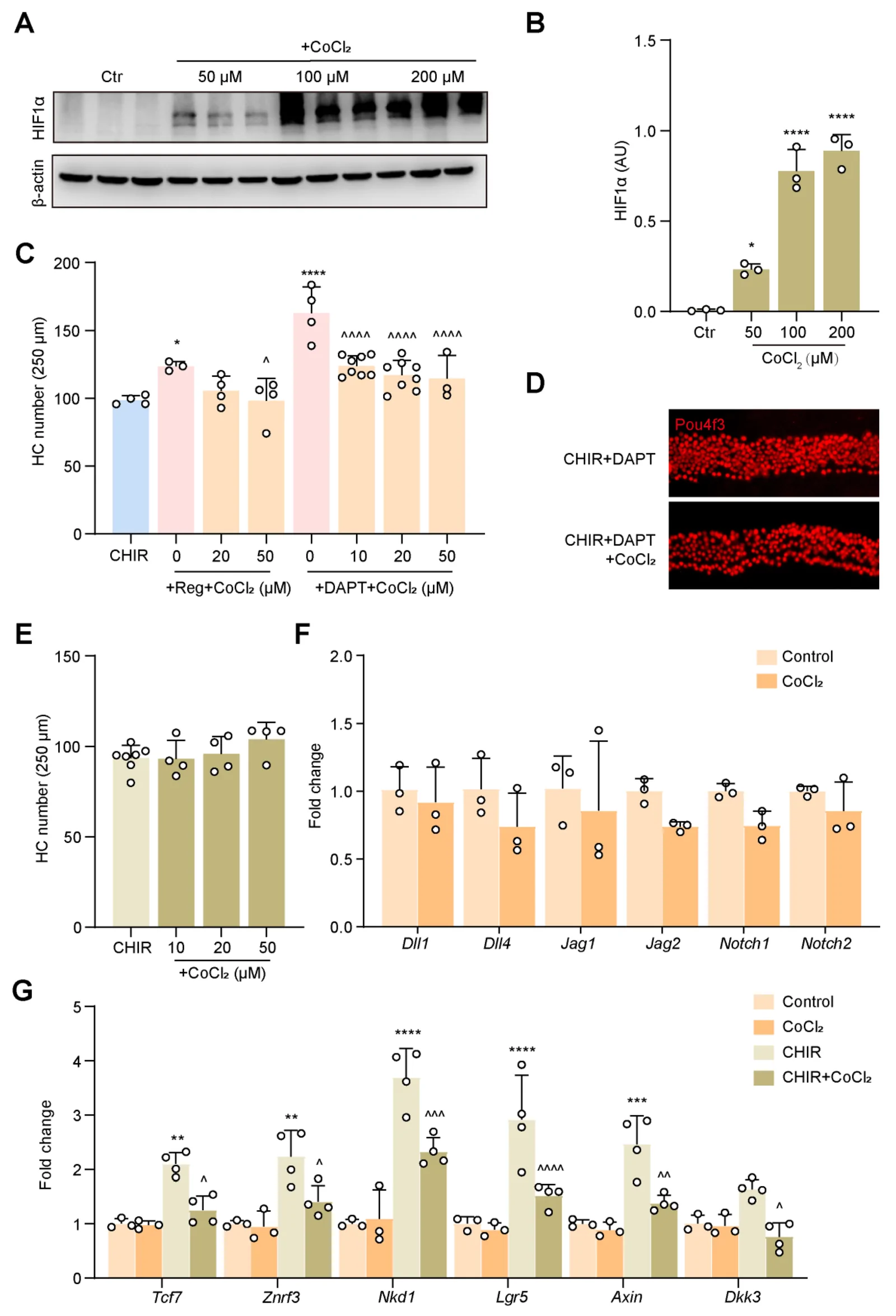
HIF1α accumulation inhibited hair cell reprogramming and affected Wnt signaling. (**A**,**B**) HIF1α accumulation was induced by CoCl_2_ in cochlear explants. Densitometric quantification of the Western blot analysis from (**A**). Error bars represent mean ± SEM. *n* = 3 independent experiments. * *p* < 0.05, **** *p* < 0.0001 by one-way ANOVA. (**C**,**D**) CoCl_2_ inhibited DAPT- or Regorafenib-induced hair cell reprogramming. Error bars represent mean ± SD. *n* = 3–8 cochlear explants in each condition. (**C**) * *p* < 0.05 and **** *p* < 0.0001 by one-way ANOVA compared with CHIR99021. ^ *p* < 0.05 and ^^^^ *p* < 0.0001 by one-way ANOVA compared with Regorafenib and DAPT. (**D**) Immunofluorescence images of cochlear explants. (**E**) CoCl_2_ did not affect hair cell survival. Error bars represent mean ± SD. *n* = 4–7 cochlear explants in each condition. (**F**) RT-qPCR analysis revealed that CoCl_2_ did not affect the Notch signaling pathway. Error bars represent mean ± SD. *n* = 3 cochlear explants in each condition. (**G**) RT-qPCR analysis revealed that CoCl_2_ affected the Wnt signaling pathway. Error bars represent mean ± SD. *n* = 3–4 cochlear explants in each condition. ** *p* < 0.01, *** *p* < 0.001 and **** *p* < 0.0001 by one-way ANOVA compared with control. ^ *p* < 0.05, ^^ *p* < 0.01, ^^^ *p* < 0.001 and ^^^^ *p* < 0.0001 by one-way ANOVA compared with CHIR99021.

To directly test whether HIF1α accumulation is responsible for the observed impairment, we treated cochlear explants with CoCl_2_, a chemical inducer of HIF1α stabilization. Western blot analysis confirmed that CoCl_2_ treatment induced HIF1α accumulation in cochlear explants (Figure 3A,B). Having established HIF1α accumulation, we next assessed its functional impact on HC reprogramming. CoCl_2_ treatment significantly inhibited DAPT- or Regorafenib-induced hair cell reprogramming (Figure 3C,D). To rule out the possibility that this effect was secondary to cell loss, we confirmed that CoCl_2_ did not affect hair cell survival (Figure 3E). We then asked which signaling pathways might mediate this inhibitory effect. RT-qPCR analysis revealed that CoCl_2_ did not affect the Notch signaling pathway (Figure 3F). However, CoCl_2_ significantly affected the Wnt signaling pathway (Figure 3G). Compared with the control, CoCl_2_ treatment altered the expression of Wnt-related genes, and compared with CHIR99021, CoCl_2_ co-treatment resulted in the differential expression of these genes. These findings suggest that HIF1α accumulation inhibits hair cell reprogramming at least in part through the modulation of Wnt signaling rather than through effects on the Notch pathway.

### 3.4. Pharmacological Inhibition of HIF1α Rescues Hair Cell Reprogramming Under Hypoxia

**Figure 4:**
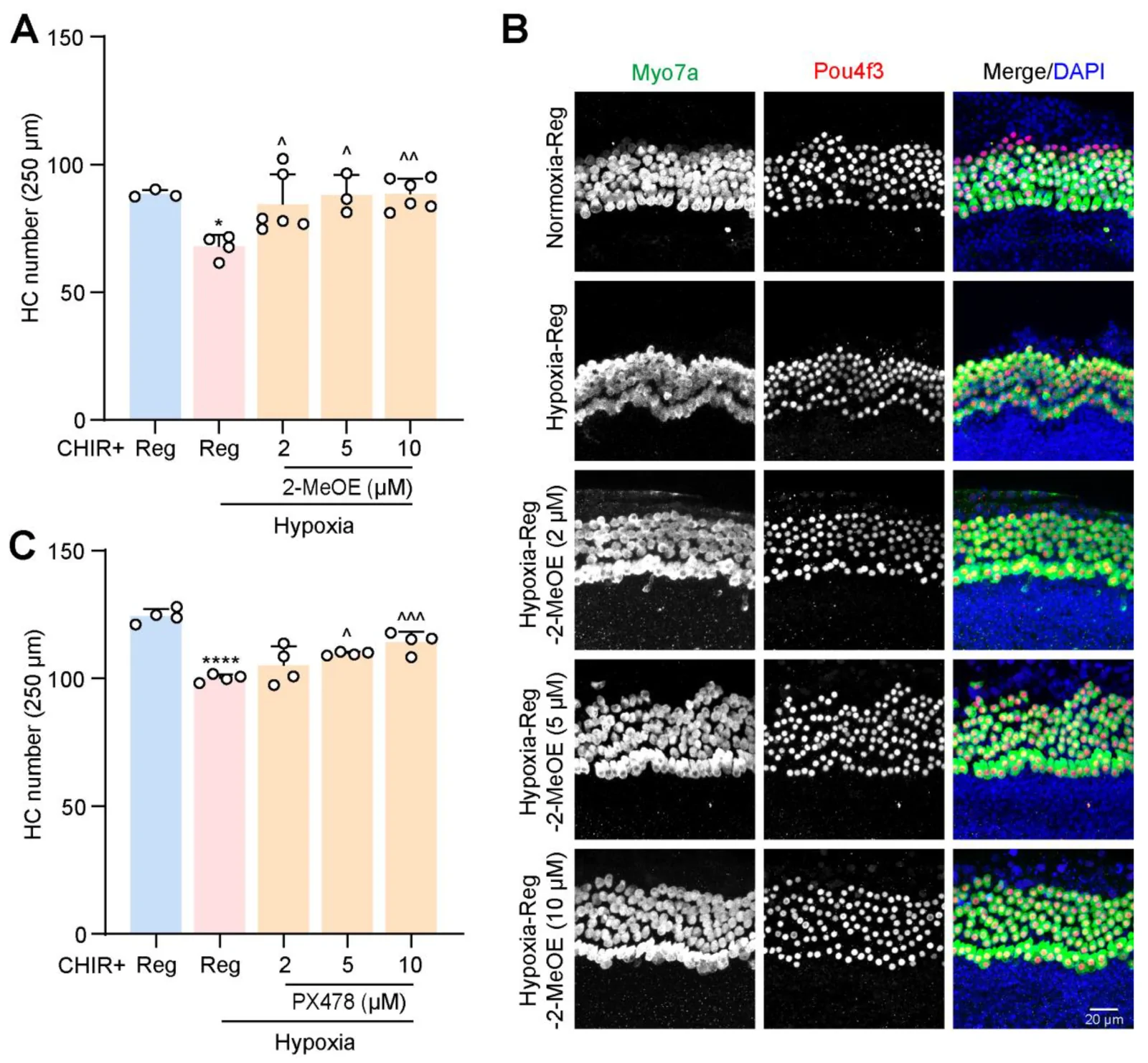
HIF1α inhibitor rescued impaired hair cell reprogramming under hypoxia conditions. (**A**) Hair cell counts in cochlear explants treated with Regorafenib alone or together with 2-MeOE (a HIF1α inhibitor). Error bars represent mean ± SD. *n* = 3–6 cochlear explants in each condition. * *p* < 0.05 by one-way ANOVA compared with normoxia. ^ *p* < 0.05 and ^^ *p* < 0.01 by one-way ANOVA compared with co-treatment with 2-MeOE in hypoxia. (**B**) Confocal images of cochlear explants from (**A**). (Scale bars: 20 μm.) (**C**) Hair cell counts in cochlear explants treated with Regorafenib alone or together with PX478 (a HIF1α inhibitor). Error bars represent mean ± SD. *n* = 4 cochlear explants in each condition. **** *p* < 0.0001 by one-way ANOVA compared with normoxia. ^ *p* < 0.05 and ^^^ *p* < 0.001 by one-way ANOVA compared with co-treatment with PX478 in hypoxia.

To determine whether HIF1α inhibition could overcome the reprogramming block induced by hypoxia, we treated cochlear explants with Regorafenib alone or together with HIF1α inhibitors (2-MeOE or PX478) under both normoxic and hypoxic conditions. Treatment with 2-MeOE, a HIF1α inhibitor, partially rescued the impaired hair cell reprogramming induced by hypoxia in Regorafenib-treated cochlear explants. Quantification showed that hypoxia significantly reduced hair cell numbers compared to normoxia, while co-treatment with 2-MeOE in hypoxia significantly restored hair cell reprogramming (Figure 4A). Confocal imaging confirmed the protective effect of 2-MeOE on hair cell reprogramming under hypoxic conditions (Figure 4B). To exclude compound-specific effects and strengthen the pharmacological evidence, we repeated the rescue experiment using PX478, another HIF1α inhibitor, which similarly restored hair cell reprogramming under hypoxic conditions (Figure 4C).

Together, hypoxia significantly reduced Regorafenib-induced hair cell reprogramming compared to normoxia, while co-treatment with 2-MeOE or PX478 in hypoxia significantly restored hair cell numbers. These results demonstrate that pharmacological inhibition of HIF1α can rescue the reprogramming block imposed by hypoxic conditions, suggesting that HIF1α may represent a potential therapeutic target for promoting hair cell regeneration, though further in vivo validation is required.

## 4. Discussion

In this study, we identify HIF1α accumulation as a previously underappreciated barrier to hair cell (HC) reprogramming from supporting cells in the mammalian cochlea. Our findings demonstrate that the cochlear hypoxic niche stabilizes HIF1α, which specifically antagonizes Wnt-mediated HC reprogramming without affecting supporting cell survival. This work provides a mechanistic explanation for the long-observed disconnect between in vitro efficacy and in vivo failure and highlights HIF1α as a druggable target for hearing restoration.

The observation that HIF1α accumulates in the basilar membrane in vivo but is rapidly lost upon explant culture suggests that oxygen tension is a primary determinant of regenerative competence. Physiological hypoxia (2–5% O_2_) is characteristic of the inner ear [11], and our data show that recapitulating this oxygen level in vitro or chemically stabilizing HIF1α with CoCl_2_ effectively blocks HC reprogramming. Importantly, hypoxia did not reduce HC survival, which distinguishes the effect from generalized cytotoxicity and points to a specific inhibition of the reprogramming program.

Mechanistically, we found that HIF1α suppresses Wnt signaling rather than Notch signaling. While Notch inhibition by DAPT or Regorafenib remained effective in promoting HC reprogramming under normoxia, its effects were markedly attenuated under hypoxia or CoCl_2_ treatment. RT-qPCR analysis confirmed that HIF1α stabilization altered the expression of Wnt-related genes, including *Wnt10a*, without affecting Notch components. This is consistent with reports in other stem cell systems where HIF1α intersects with β-catenin signaling, often through direct transcriptional repression or competition for co-activators [14,16]. The selective suppression of Wnt may explain why approaches relying heavily on Wnt activation are particularly vulnerable to the in vivo microenvironment.

Crucially, pharmacological inhibition of HIF1α with 2-MeOE or PX478 rescued HC reprogramming under hypoxia, restoring the efficacy of Regorafenib-based protocols. Both compounds are well-characterized HIF1α inhibitors with distinct mechanisms: 2-MeOE destabilizes HIF1α by disrupting microtubule dynamics, while PX478 inhibits HIF1α translation [19,20]. Their ability to overcome the hypoxic block supports the notion that HIF1α is not merely a passive marker of the in vivo state but also an active suppressor of regenerative potential. These findings have immediate translational implications, as both inhibitors have been evaluated in clinical trials for oncology, offering established safety and pharmacokinetic profiles that could accelerate repurposing for inner ear therapies [21].

Beyond the cochlear system, our findings contribute to a growing understanding of how oxygen tension and HIF1α regulate cell fate reprogramming across diverse stem and progenitor cell populations. Cellular reprogramming requires profound metabolic remodeling, and HIF1α has emerged as a central orchestrator of this process through its regulation of the glycolytic shift that reconfigures the epigenetic landscape for fate transitions [16,22,23]. Importantly, HIF1α functions as a conserved regulator of cellular plasticity, as demonstrated in neural stem cells, where HIF1α maintains quiescence and prevents premature differentiation [13]. Our demonstration that HIF1α suppresses Wnt-driven supporting cell-to-hair cell conversion in the cochlea extends this paradigm by positioning HIF1α as a gatekeeper of cellular plasticity, whose modulation could unlock the reprogramming competence of otherwise refractory cell populations. The broader implications extend beyond the auditory system, as HIF1α intersects with Wnt/β-catenin signaling across multiple tissue types, which suggests that the pharmacological targeting of HIF1α may represent a general strategy to enhance reprogramming efficiency in diverse regenerative settings [14,24]. Thus, our study not only uncovers a mechanistic barrier to hair cell regeneration but also establishes the cochlea as a tractable model for investigating how the niche microenvironment orchestrates cell fate decisions during reprogramming, with implications for stem cell biology and regenerative medicine more broadly.

## 5. Limitations

Several limitations of this study should be acknowledged, and several important questions remain. First, the sample size of the in vivo experiments was small (*n* = 3 per experiment), and our findings are currently confined to a single strain of neonatal mice. Therefore, verification in larger cohorts and in other mouse strains, as well as in adult mice and guinea pigs, is essential, particularly because neonatal mice possess a regenerative capacity that declines with age [2]. Second, the reprogramming of hair cells, albeit relatively small in number, was confirmed primarily by immunofluorescence of hair cell markers; other assays such as FACS, while technically challenging, should provide further support to the induced cellular reprogramming. These constraints limit the interpretation of regenerative potential and cellular phenotypes. Third, several mechanistic questions remain unresolved. For example, the specific supporting cell subtype(s) whose reprogramming is gated by HIF1α remain to be identified. While our organoid and explant systems recapitulate the behavior of the broader supporting cell population, lineage-tracing experiments using subtype-specific drivers would clarify whether HIF1α-enforced quiescence is uniformly distributed or concentrated in specific progenitor subpopulations. Moreover, while our pharmacological data strongly implicate HIF1α as a suppressor of Wnt signaling, we cannot definitively establish a direct causal relationship without genetic manipulation (e.g., HIF1α knockdown or overexpression in conjunction with Wnt inhibition). The precise molecular mechanism by which HIF1α suppresses Wnt10a expression in this context warrants further investigation. Whether it occurs through direct promoter binding, histone modification, or indirect regulation via upstream modulators remains unresolved. Additionally, whether the metabolic shift from OXPHOS to glycolysis under hypoxia independently contributes to the reprogramming blockade and whether metabolic normalization synergizes with HIF1α inhibition represent important avenues for future work. Finally, the therapeutic potential of HIF1α inhibition in adult or injured cochleae, as opposed to neonatal explants and organoids, will require validation in clinically relevant in vivo models, a critical step toward translation. Addressing these questions will deepen our mechanistic understanding, while the current findings already provide a sufficient foundation for therapeutic translation.

## 6. Conclusions

Our study positions HIF1α as a central node linking the cochlear hypoxic microenvironment to HC reprogramming failure. By demonstrating that HIF1α suppresses Wnt-dependent HC reprogramming and that its inhibition rescues this block, we provide a rational framework for combining HIF1α antagonists with established regenerative agents. This strategy may offer a clinically viable path to overcome the intrinsic barriers to HC regeneration and restore hearing function.

## Data Availability

The original contributions presented in this study are included in the article/Supplementary Material. Further inquiries can be directed to the corresponding authors.

## Supplementary Materials

The following supporting information can be downloaded at: https://www.mdpi.com/article/10.3390/cells15161440/s1, Figure S1. Experimental Design Overview. Table S1. Details of reagents, antibodies and primers used in the study.
