# Supplementary material for: Hypoxia-Stabilized HIF1α Restricts Hair Cell Reprogramming by Suppressing Wnt Signaling in the Mammalian Cochlea
Source: Cells. 2026 Aug 11;15(16):1440. doi: 10.3390/cells15161440 (PMC13510866; doi:10.3390/cells15161440)

Table S1. Details of reagents, antibodies and primers used in the study.

Key resources: regents				
**Reagent Name**	**Abbreviation**	**Final concentration**	**Vendor**	**Cat#**
Murine EGF	E	50 ng/ml (passage 1)<br>25 ng/ml (passage 2)	Peprotech	315-09
Murine FGF-basic	F	50 ng/ml	Peprotech	450-33
Murine IGF-1	I	50 ng/ml	Peprotech	250-19
CHIR99021	CHIR/C	3 μM (passage 1)<br>2 μM (passage 2)	Selleck	S1263
Valproic Acid Sodium Salt	VPA/V	1 mM	Sigma	676380
2-phospho-L-ascorbic acid	pVc/P	100 μg/ml	Sigma	49752
616452	6	2 μM	Selleck	S7223
Deshydroxy LY411575	LY	10 μM	Santa Cruz	209984-56-5
Regorfenib	Reg	1, 10 μM	Selleck	S1178
DAPT	D	5 μM	Santa Cruz	sc-201315
Advanced DMEM/F12	-	-	Gibco	12634010
B-27 Supplement W/O	-	-	Gibco	12587010
N2 supplement	-	-	Gibco	17502048
Glutamax I	-	-	Gibco	35050061
Penicillin G	-	2.4 mg/ml	Sigma	P3032

Laminin	-	20 ng/ml	Corning	354232
Geltrex	-	2%	Yeasen	40191ES08
(z)-4-Hydroxytamoxifn	-	10 μM	Tocris	3412
Thermolysin	-	1 mg/ml	Sigma	P1512
TrypLE	-	-	Thermo	12604013
DNAse I	-	200 U/ml	Sigma	AMPD1
2-Methoxyestradiol	2-Meoe	2，5，10μM	Selleck	S1233
PX-478 2HCl	PX478	2，5，10μM	Selleck	S7612
Key resources: antibodies and fluorescent molecules				
**Antibody**	**Dilution**	**Source**	**Vendor**	**Cat#**
488-affinipire goat anti-rabbit IgG	1:500	Secondary antibodies	Jackson	111-545-003
594-affinipire goat anti-mouse IgG	1:500	Secondary antibodies	Jackson	115-585-003
Myosin VIIa	1:500	Rabbit Polyclonal	Proteus Biosciences	25-6790
Pou4f3	1:200	Mouse monoclonal	Santa Cruz	sc-81980
β-Actin	1:1000	Rabbit monoclonal	Cell Signaling Technology	4970
DAPI	1:2000	Fluorescent labeled nucleus	Roche	28718-90-3

HIF1α	1：1000	Rabbit monoclonal	Cell Signaling Technology	36169
Key resources: qPCR primers				
**Gene**	**Forward primer**	**Reverse primer**		
Idh2	AACACCGACGAGTCCATT	GTCAGTCTTATAGTGCTTGTCAA		
Sdha	GACATCAAGACTGGCAAGGTTA	GAGCGGATAGCAGGAGGTA		
Tcf7	ACAGTGCTCTAGGCTGTCC	CGACCTGAGAATGTTGGTGCT		
Znrf3	GGAGACAAGCAACCTTAC	GATGGCATTGGTCCTATG		
Nkd1	GAGGACGAGGACGACTTC	CCATTCTCTTCTCATCTCCACTA		
Lgr5	GAGACACTAGACCTGAACT	TACTGGAATGCTGACCTT		
Flt1	CATCGGCAGACCAATACA	GCAGTTGAGGACAAGAGT		
Igf2	GGACGTGTCTACCTCTCA	GGTGTCATATTGGAAGAACTTG		
Nos1	CCATCGTGTCCTACCATA	CATCGTCAGCCTGTATTC		
Pou5f1	CCGTGTGAGGTGGAGTCT	CTGATTGGCGATGTGAGTGAT		
PDK1	CGACAGACTCTTCAACTA	GAGCCTTAATGTAGATAACC		
Pfkm	GGCTCTCGTCTCAACATCATCATT	CGCTTCACCACCAGGTTCTT		
HK1	GGTCACGATGTAGCCACTT	AGTCCAATCTCACAAGAAGGTT		
Wnt10a	AACGCTTCTCTAAGGACTTTCTGGA	CGGTTGTTGTGGAGTCTCATTCG		
Pou4f3	TCTGGCGGCGGTGGATAT	GCTGCTCATGGTATGGTAGGT		
Myo7a	GGATGTGCGAGATGCCTTTG	GCTTGTAGATTGCTGCGTTGA		
Dll1	GGCTTCTCTGGCTTCAACT	CACTTGGCACCGTTAGA		
Jag1	GGACGGAGACAACTGGTA	GGAAGACTGGCACTCATTG		

Eno1	GAACTCCGAGACAATGATAAGA	TGCTCCACAACATTCACTT
Eno2	GGCACTCTACCAGGACTT	GTTGGCTGTGAACTTGGA
Ndufa5	CGACACTCCACACGAGAG	GCTCCGCCTTGACCATAT
Dll4	GAACCTTCTCACTCAACAT	GAGCCTTGGATGATGATT
Jag2	TATAACGGAGGCATCTGT	CTGGCACTCATCAATGTT
Notch1	ATCAATGAGTTCCAATGTC	CCTGTGTAACCTTCTGTA
Notch2	GTATCTCCAAGCCGTGTA	GTCCTCTTCACAGTCCAT
Axin2	CACCAAGACCTACATACGA	CCACTCCTCACATATTCCA
Dkk3	GCTTACCTCCCAACTATCA	CTCCTCTTGCCTTCTTCA
Aldh1a1	TAGCAGCAGGAGTCTTCAC	ACAGCCAAATAGCAGTTCAC

Figure S1. Experimental Design Overview

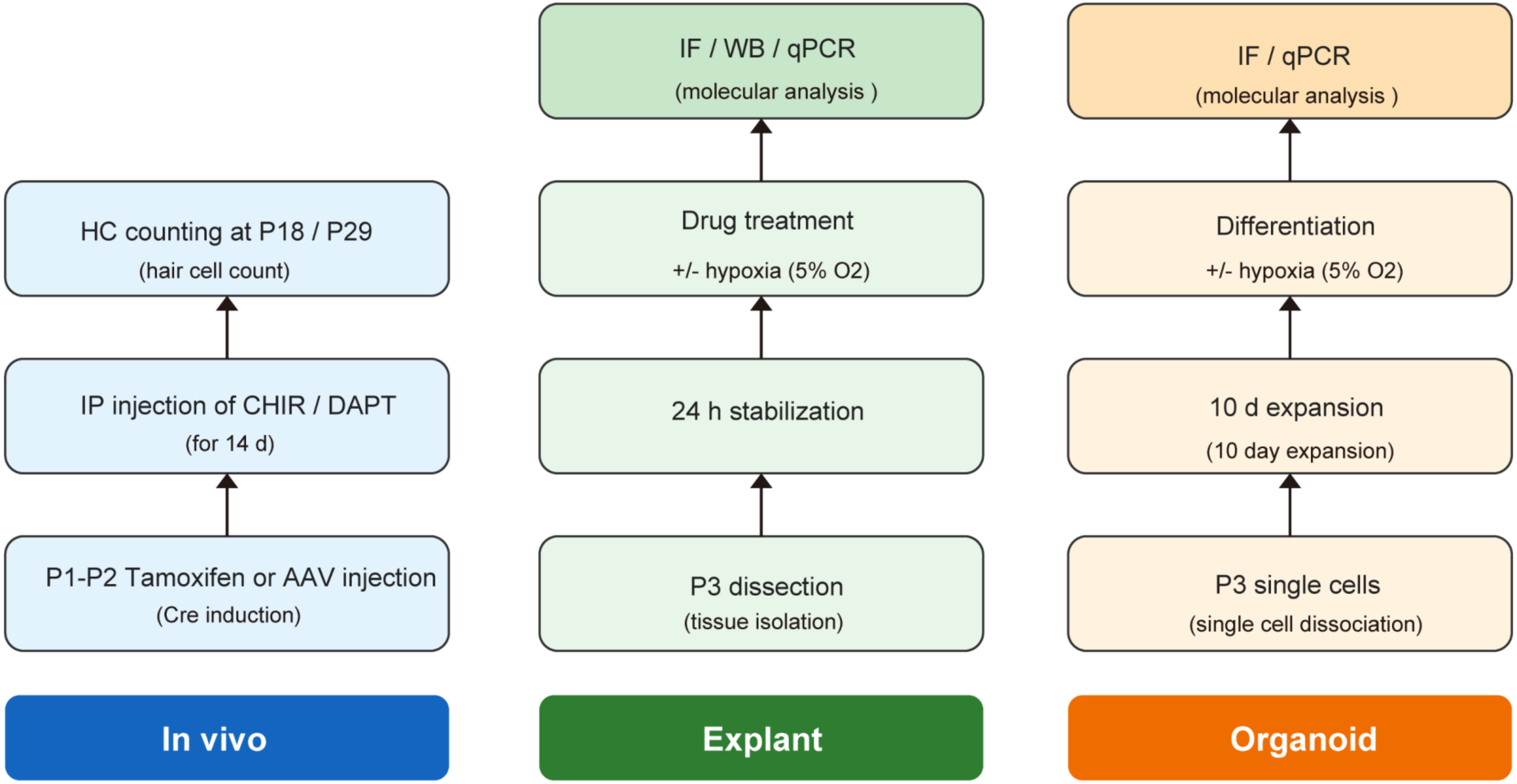

Supplement: Supplementary file 1 [file cells-15-01440-s001.zip › cells-4452337-supplementary.pdf]
